# Dark Adaptometry and Optical Coherence Tomography Angiography in Huntington Disease

**DOI:** 10.18502/jovr.v19i1.15422

**Published:** 2024-03-14

**Authors:** Aaditya Shah, Spencer Fuller, Susan Criswell, Rajendra S. Apte

**Affiliations:** ^1^Department of Ophthalmology and Visual Sciences, Washington University School of Medicine, St. Louis, MO, USA; ^2^John A. Moran Eye Center, University of Utah School of Medicine, Salt Lake City, UT, USA; ^3^Department of Neurology, Washington University School of Medicine, St. Louis, MO, USA; ^4^Department of Neurology, Barrow Neurological Institute, Phoenix, AZ, USA; ^5^Department of Ophthalmology and Visual Sciences, Washington University School of Medicine, St. Louis, MO, USA

**Keywords:** Ophthalmology, Retina, Imaging, Neurology

## Abstract

**Purpose:**

Huntington's Disease (HD) is a fully penetrant neurodegenerative disease leading to cognitive and motor disturbances. The retina may serve as a structural and functional extension of the central nervous system to identify biomarkers of HD using noninvasive imaging technology such as optical coherence tomography angiography (OCTA) and dark adaptometry.

**Methods:**

This case–control study included 12 HD participants (24 eyes) recruited from the Huntington's Disease Society of America Center of Excellence at Washington University in St. Louis along with 16 control participants (31 eyes). Disease-positive participants underwent imaging testing of retinal capillary density and foveal avascular zone utilizing OCTA along with dark adaptometry testing. Data were collected from November 2020 to February 2022.

**Results:**

Individuals with HD had a lower mean age-adjusted superficial foveal capillary density and a higher mean deep foveal capillary density compared to control subjects. There was no significant difference in the mean foveal avascular zone or in dark adaptometry testing between the two groups.

**Conclusion:**

This study suggests that changes in retinal biomarkers may exist in patients with HD and that additional investigations using multimodal techniques are warranted.

##  INTRODUCTION

Neurodegenerative diseases have long become an increasing area of interest, particularly as the global population ages. Disease understanding, however, has had inherent limitations due to the accessibility problems of studying central nervous system (CNS) tissue. The retina shares embryonic origin with the brain, specifically at the embryonic diencephalon.^[1]^ This allows the neuroretina to serve as a unique window to provide insight into deeper brain tissues as well. It has also long been believed that there is significant homology between retinal and cerebral microvasculature.^[2,3]^ Thus, there has been growing focus on analyzing the vascular and neural changes of the retina and choroid in neurodegenerative brain diseases, especially with the growth of new modalities such as optical coherence tomography angiography (OCTA).^[4,5,6,7,8,9]^

OCTA is a functional extension of optical coherence tomography (OCT) technology utilized to visualize the retinal and choroidal vasculature.^[10]^ OCT uses an infrared laser to measure the amplitude and delay of reflection to measure the depth and reflectivity of structures. These measurements comprise an axial scan, which can be sequentially combined to generate a B-scan (or cross-sectional image). Multiple B-scans acquired sequentially provide volumetric information, and these images can be compared on a pixel-by-pixel basis such that the observed signal changes can be attributable to erythrocyte motion. Algorithms within the OCTA software are then utilized to create a full-thickness retinal angiogram with images segmented into superficial and deeper layers.^[11,12]^

Dark adaptation testing measures the rate at which the rod and cone system recover sensitivity in the dark following exposure to a bright light source. Although impairments have been seen with increased age, worsened visual acuity, and retinal diseases such as age-related macular degeneration, the effects of brain neurodegenerative diseases on dark adaptation are not completely elucidated.^[13]^

Huntington's Disease (HD) is a neurodegenerative disease with an autosomal dominant inheritance pattern related to a mutation in the HTT gene. It is fully penetrant and can lead to cognitive and motor disturbances.^[14]^ Although changes in visuospatial processing and visual perception have been studied previously, few studies have utilized OCT technology to study structural changes in the retina in those with HD. Parameters such as temporal retinal nerve fiber layer (RNFL) thickness, macular RNFL thickness, and ganglion cell layer (GCL) thickness have been found to be lower in those with HD compared to controls.^[15,16,17]^

To the best of our knowledge, no study to date has analyzed dark adaptometry testing in HD patients and a single study using OCT angiography was recently published that found no statistically significant difference between HD patients and controls for superficial capillary plexus density, deep capillary plexus density, and choriocapillaris density.^[18]^

##  METHODS

Study participants were recruited from Washington University in St. Louis, specifically through the Huntington's Disease Society of America Center of Excellence. The study design was approved by the institutional review board of Washington University in St. Louis at the Human Research Protection Office and adhered to the tenets of the Declaration of Helsinki. Risks and benefits were discussed with each participant, and informed consent was obtained before beginning the ophthalmic examination and testing.

Data for this study were collected from November 2020 until February 2022. Inclusion criteria required a Unified Huntington's Disease Rating Scale-Total Motor Score (UHDRS-TMS) and gene-positive testing for the *HTT *gene. The UHDRS-TMS rates various motor signs including eye movements, speech, chorea, dystonia, bradykinesia, and gait in order to provide a measure of motor symptom severity.

Exclusion criteria included a known history of previous retinal diseases including age-related macular degeneration and diabetic retinopathy or any retinal disease that may have required intraocular surgery; intraocular pressure of 22 mmHg or higher; and previous retinal laser therapy.

Control data was acquired from individuals without known Huntington Disease who otherwise had the same exclusion criteria as those above. This control group has been reported on in a previous study.^[5]^

**Figure 1:**
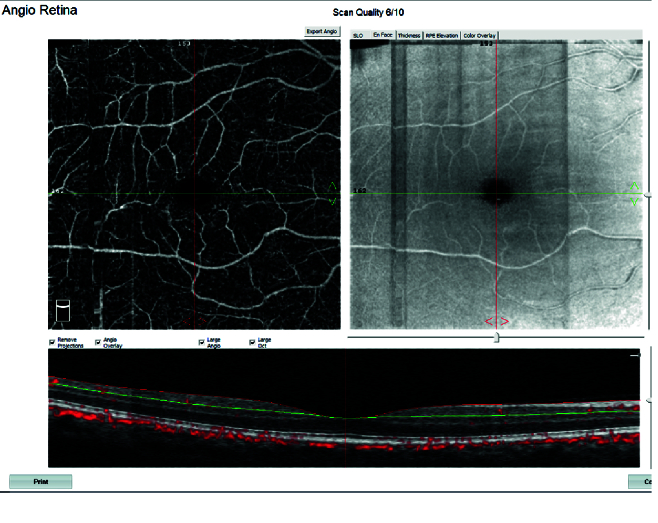
Optovue OCTA image of macula with superficial retinal capillary plexus overlay.

**Figure 2:**
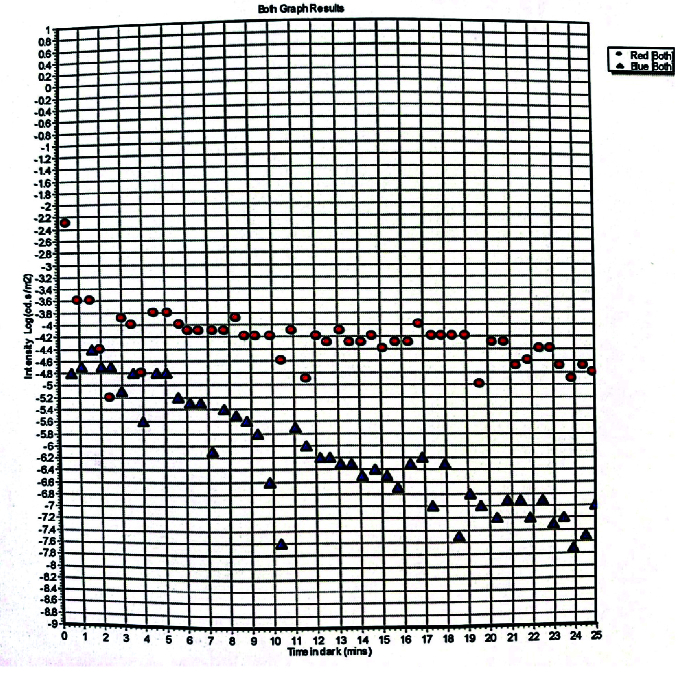
Representative Diagnosys dark adaptometry image.

All participants received an ophthalmic exam that consisted of Snellen visual acuity assessment, ocular motility testing, intraocular pressure measurements, and examination of the anterior segment and dilated fundus. OCTA imaging of the macula was performed using the Avanti Optovue OCTA system (Optovue, Visionix USA) as shown in Figure 1. Measurements were automated using the manufacture's software (Optovue RTVue) providing an objective manner of collection. Data outcomes collected into superficial vessel capillary density at the fovea, deep vessel capillary density at the fovea, and foveal avascular zone (FAZ). Additionally, dark adaptometry testing was performed using the Diagnosys Dark Adaptometry system to measure cone sensitivity, rod sensitivity, and the rod-cone break point as shown in Figure 2. Measurements were automated using the manufacture's software providing a plot of the results.

### Data Analysis

Foveal avascular zone, superficial foveal capillary density, and deep foveal capillary density in patients with HD were compared to controls using two sample *t*-tests with equal variances. Further, linear regression was performed and adjusted for age, a known confounder for measurements of the retinal vasculature.^[19]^ Statistical analysis was performed using the STATA/IC software version 14.2 (StataCorp LP, College Station, TX, USA) and Microsoft Excel (2019) with alpha set at 0.05.

##  RESULTS

A total of 24 eyes from 12 individuals with HD were included in the study (mean age [SD], 52.5 [10.8]). Of these, 11 individuals were Caucasian and 1 was African American. Eight eyes were excluded based on abnormal motion artifact (due to underlying HD-related motor disease) when completing OCTA testing. All 12 individuals were included in dark adaptometry testing. Additionally, 31 control eyes were included for comparison of OCTA testing (mean age [SD], 75.4 [6.6] years). All control individuals were Caucasian. One of the control eyes was excluded due to artifact obscuring the reliability of the measurements.

**Figure 3:**
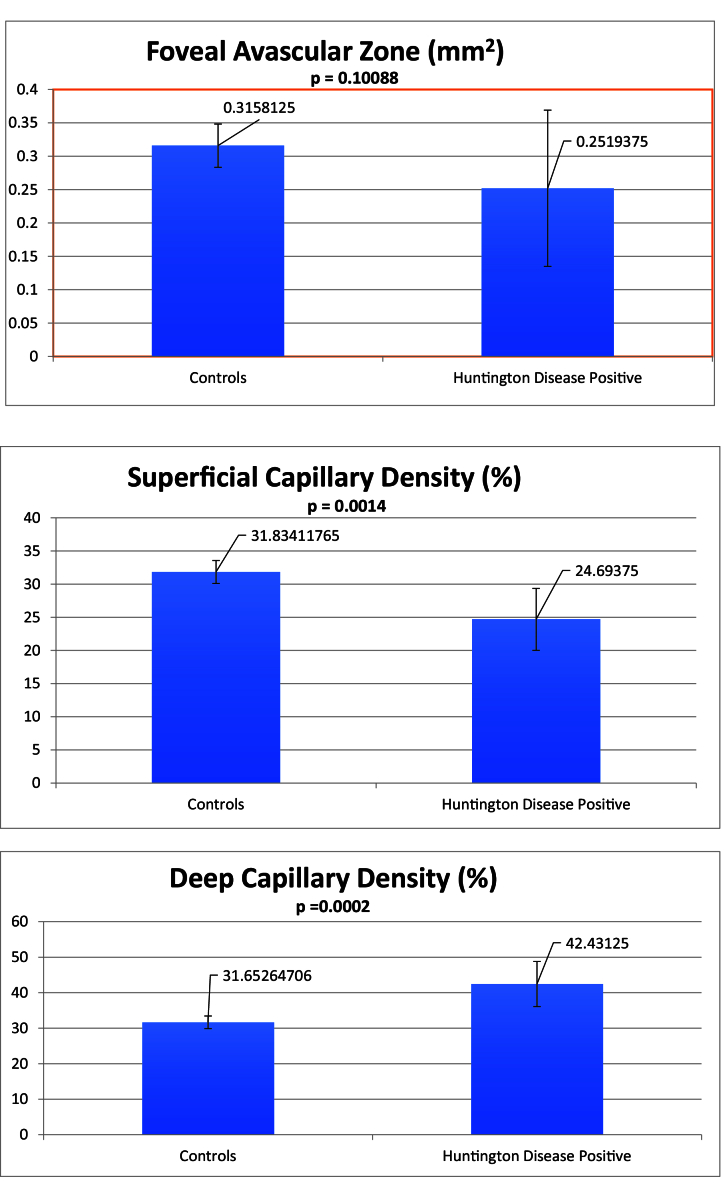
Mean foveal avascular zone, superficial foveal density, and deep foveal density measures with 95% confidence intervals.

In regard to OCTA testing, 16 eyes with HD and 31 control eyes completed the test. As shown in Figure 3, the HD eyes had a mean superficial foveal capillary density of 24.7% compared to the control eyes of 31.8% (*P* = 0.0014, SD = 5.01%). The mean deep foveal capillary density in the HD group was 42.4% compared to 31.7% in the control group (*P* = 0.0002, SD = 5.17%). The foveal avascular zone was 0.252 mm^2^ in the HD eyes compared to 0.316 mm^2^ in the control eyes (*P* > 0.05, SD = 0.09 mm^2^).

Regression analysis revealed that when accounting for age, individuals in the HD group still maintained a lower mean superficial foveal capillary density (*P* = 0.022) and a higher mean deep foveal capillary density (*P* = 0.033). This difference did not diverge with worsening HD severity measures–there was no statistically significant change in the superficial or deep foveal capillary density or the foveal avascular zone with increased severity based on the UHDRS-TMS (*P* > 0.05).

All 12 individuals with HD had dark adaptometry testing that was considered within the range of normal based on the machine-generated plot of cone sensitivity, rod sensitivity, and the rod-cone break point.

##  DISCUSSION

This study demonstrated a significant difference between the superficial and deep foveal capillary densities in those with HD and control individuals using the OCTA. This remained significant after controlling for age in our analysis. There was no significant difference in OCTA parameters with worsened disease severity (via the UHDRS-TMS score) and no significant difference in foveal avascular zone between the two groups. There has been only one other study analyzing retinal vasculature using OCTA in those with HD. In that study, there was no statistically significant difference between HD patients and controls for superficial capillary plexus density, deep capillary plexus density, and choriocapillaris density.^[18]^ Additionally, to the best of our knowledge, no other study has analyzed dark adaptometry in patients with HD; therefore, this was a unique aspect to the study. However, in our population, dark adaptometry testing was otherwise unremarkable in the HD patients. This may be for a number of different reasons. Firstly, it may take more significant disease severity to manifest changes in dark adaptation. Secondly, photoreceptor function may be generally spared in HD such that changes in dark adaptation are not seen. Thirdly, it may also be that the sample size of this study was too small to ascertain any change.

Recent studies have shown that altered neurovascular structure may be a true biomarker of HD. Lin et al showed an increase in cerebral vasculature density in post-mortem human tissue and mouse models.^[20]^ Drouin-Oullet et al reported similar findings in human tissue and mouse models as well and discussed that this may be a compensatory response to maintain proper oxygenation of tissues despite an overall decrease in the number of larger blood vessels.^[21]^ Additionally, there has been some precursory evidence for retinal dysfunction in the setting of HD. For instance, Paulus et al conducted an electrophysiologic study showing that those with HD had cone dysfunction when compared to normal controls.^[22]^ Two other recent ERG studies have demonstrated altered retinal responses in subjects with HD compared to controls.^[23,24]^

Interestingly in our study, the HD group had a lower mean superficial foveal capillary density and a high deep foveal capillary density. Similar to what was seen in post-mortem cerebral vasculature as discussed earlier, the increase in the deep foveal capillary density may be a compensatory response to hypoperfusion. The superficial foveal capillary density may on the other hand be a surrogate for true hypoperfusion at the level of the retina. Further, while retinal vasculature was the focus of this study, the retinal vascular changes may also provide insight into the blood–brain barrier (BBB) dysfunction in HD individuals especially as these mechanisms have remained elusive given the lack of in vivo analysis. In this study, there was no significant change in these variables with changes in the UHDRS-TMS score, however, further study with a larger sample size and thus a higher range of UHDRS-TMS scores will be required to understand the relationships between these variable and disease progression.

There are important limitations to be acknowledged in this study. Our sample size was small, which makes it difficult to ascertain true differences in the results. Furthermore, while the understanding of retinal involvement is limited in HD, these findings (a lower mean age-adjusted superficial foveal capillary density and a higher mean deep foveal capillary density compared to control subjects) indicate the need of further study to assess true retinal biomarkers of disease. Altogether, this study suggests that changes in retinal biomarkers may exist in patients with HD and that additional investigations using multimodal techniques are warranted.

##  Financial Support and Sponsorship

None.

##  Conflicts of Interest

None.
